# External influences and priority-setting for anti-cancer agents: a case study of media coverage in adjuvant trastuzumab for breast cancer

**DOI:** 10.1186/1471-2407-7-110

**Published:** 2007-06-28

**Authors:** Christopher M Booth, George Dranitsaris, M Corona Gainford, Scott Berry, Michael Fralick, John Fralick, Joanna Sue, Mark Clemons

**Affiliations:** 1 National Cancer Institute of Canada Clinical Trials Group, Queen's University, Kingston, Canada; 2 Princess Margaret Hospital, University of Toronto, Toronto, Canada; 3 Toronto Sunnybrook Regional Cancer Centre, University of Toronto, Toronto, Canada

## Abstract

**Background:**

Setting priorities for the funding of new anti-cancer agents is becoming increasingly complex. The funding of adjuvant trastuzumab for breast cancer has brought this dilemma to the fore. In this paper we review external factors that may influence decision-making bodies and present a case study of media response in Ontario, Canada to adjuvant trastuzumab for breast cancer.

**Methods:**

A comprehensive search of the databases of Canadian national and local newspapers and television was performed. Articles pertaining to trastuzumab in adjuvant breast cancer as well as 17 other anti-cancer drugs and indications were retrieved. The search period was from the date when individual trial results were announced to the date funding was made available in Ontario.

**Results:**

During the 2.6 months between the release of the trastuzumab results to funding approval in Ontario, we identified 51 episodes of media coverage. For the 17 other drugs/indications (7 breast and 10 non-breast), the median time to funding approval was 31 months (range 14–46). Other recent major advances in oncology such as adjuvant vinorelbine/cisplatin for resected NSCLC and docetaxel for advanced prostate cancer received considerably less media attention (17 media reports for each) than trastuzumab. The median number of media reports for breast cancer drugs was 4.5 compared to 2.5 for non-breast cancer drugs (p = 0.56).

**Conclusion:**

Priority-setting for novel anti-cancer agents is a complex process that tries to ensure fair use of constrained resources to fund therapies with the best evidence of clinical benefit. However, this process is subject to external factors including the influence of media, patient advocates, politicians, and industry. The data in this case study serve to illustrate the significant involvement one (or all) of these external factors may play in the debate over priority-setting.

## Background

With the number and cost of new anti-cancer drugs rising dramatically, setting priorities for funding these therapies is becoming increasingly complex in private and public health care systems worldwide. The role of trastuzumab in the adjuvant management of breast cancer has brought this dilemma to the fore.

Criteria for establishing a legitimate and fair process for priority-setting have been proposed by Daniels and include: "...transparency about the grounds for decisions; appeals to rationales that all can accept as relevant to meeting health needs fairly; and procedures for revising decisions in light of challenges to them." [1].

Two studies have specifically evaluated the process of rationing new anti-cancer therapies. In their review of decision-making at Christie Hospital NHS Trust (UK), Foy et al described that funding decisions were based largely on evidence thresholds which were cut-off points determined from information on effectiveness [2]. In a study of priority-setting decisions for new cancer drugs in Ontario, Canada Martin and colleagues reported that although clinical benefit was the primary factor in decision-making, rationales could change with changing costs and/or budgets [3]. Common to both of these studies was the finding that external factors including pressure from political, social and physician groups had some role in decision-making.

Media coverage of health is recognized as one external factor which may influence the delivery of health care. A recent Cochrane Library overview found that mass media information on health-related issues may induce changes in health services utilization, both through planned campaigns and unplanned coverage [4]. The manner in which the media portray an issue can have significant implications on the public's participation in rationing of health care resources. This was seen in the media coverage of the Child B case in the UK when a young child was denied funding for further chemotherapy and a second bone marrow transplant for advanced leukemia. Data from this controversy depicted how different media outlets can portray the same story in vastly different lights [5].

**Figure 1:**
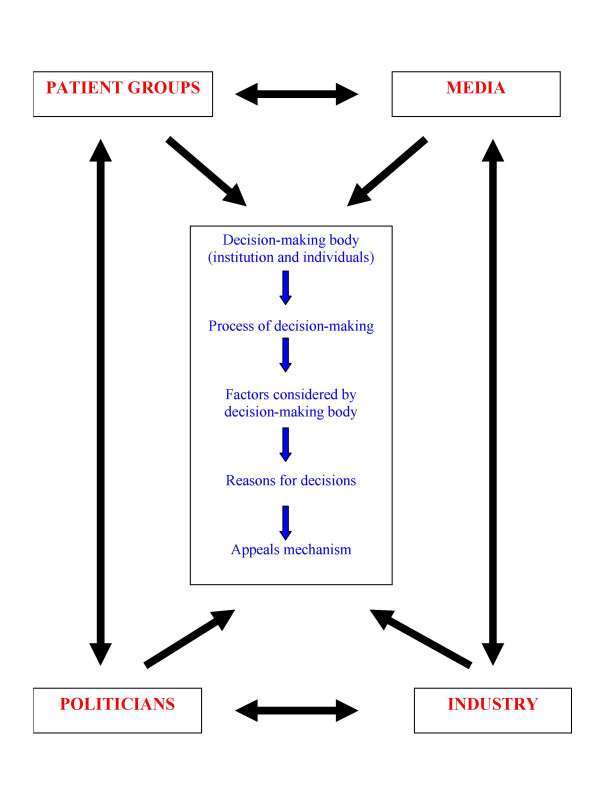
Potential interaction between external factors (shown in red) and the domains of priority-setting (shown in blue).

The recent process of approving funds for adjuvant trastuzumab in breast cancer in Ontario and the UK has highlighted the need for a legitimate and fair approach to priority-setting. It has also brought to the forefront the potential influence of external factors such as patient groups, politicians, the media, and industry. In a recent commentary piece Ferner and McDowell [6] described how several factors may influence the National Institute of Health and Clinical Excellence (NICE) in its appraisal of the evidence for adjuvant trastuzumab in breast cancer. The potential for such external factors to interact with each other and with proposed domains of priority-setting [7] are shown in Figure 1.

While external factors are recognized as having potential to influence priority-setting, there is a paucity of data describing specific examples of how such factors can manifest in the rationing of anti-cancer therapy. In this paper we present a case study which contrasts the Ontario media's response to adjuvant trastuzumab with other anti-cancer agents to provide a detailed example of how one of these external factors (i.e. media coverage) may influence decision-making bodies. For comparative purposes we also evaluated the degree of media interest for 17 other novel anti-cancer agents and indications. The second objective of this study was to describe time to funding for each of these anti-cancer agents.

## Methods

### Case study: process of drug funding in Ontario

The province of Ontario has a "Committee to Evaluate Drugs" (CED) that provides specialized advice to the Ministry of Health and Long-Term Care on which therapies should be funded by the publicly funded drug program in Ontario. In 2005, an expert sub-committee of the CED was created in which members of the CED and representatives from Cancer Care Ontario (CCO – the provincial body that coordinates cancer care) consider which new cancer medications will be funded in Ontario. The CED-CCO sub-committee considers rigorously developed evidence-based guidelines from the CCO program in evidence-based care, pharmacoeconomic data, and other relevant information before making a funding recommendation to the CED. The CED reviews this recommendation and makes a final recommendation on funding to the Ministry of Health and Long-Term Care. This process ensures that cancer drugs are considered through a review mechanism that is consistent with the process used to make funding decisions for other provincially funded medications. The CED-CCO sub-committee has some major differences from the decision-making body it replaced (the CCO Policy Advisory Committee) in that its deliberations are now confidential and economic analyses are now a major component of the decision-making process.

### Case study: funding of adjuvant trastuzumab in Ontario

All four randomized trials of trastuzumab in early stage HER-2 positive breast cancer have demonstrated significant benefits for treatment with trastuzumab [8-10]. These data show a 50% relative risk reduction in breast cancer recurrence, that occurs regardless of when (concurrent versus sequential with chemotherapy) or to which type of chemotherapy regimen trastuzumab is added. More recent analyses have also demonstrated actual survival benefits [11]. Following the initial presentation of the results of three of these trials [12,13] at the American Society of Clinical Oncology (ASCO) annual meeting on May 16, 2005, there was a massive call from oncologists, patients and support groups alike for rapid access to the drug. In Ontario, trastuzumab was approved for use in early stage breast cancer at an unprecedented rate; funding approval was granted within 67 days of the ASCO presentation.

### Media database search and analysis

Time to funding approval was calculated for trastuzumab and a comparison cohort of other anti-cancer agents and indications. The comparison cohort comprised all therapies which had been submitted to Cancer Care Ontario for consideration of funding since 2000 (n = 17). Time to funding was defined as the number of months which elapsed from the release of initial study results, until the date that funding was made available in Ontario. The search was censored at August 1^st ^2005 (the start date of funding for adjuvant trastuzumab). For drugs in which a decision had not yet been reached, we report the amount of time from release of results until August 1^st ^2005.

A comprehensive search of the Toronto Public Library database was undertaken for episodes of media coverage related to each of the 18 therapeutics and indications. The search period for the media database portion of this study was defined as described for the time to funding calculation. The database includes articles from Canada's two national newspapers and major newspapers from all cities across Ontario with populations of ≥ 250,000. Broadcast news reports (television and radio) were identified using databases for the major Canadian televised news networks and FP INFOMART. The newswire website was searched for releases by Canadian Press. The final data were presented descriptively as means, medians and proportions. Spearman's rho was used to calculate the correlation between intensity of media coverage and time to drug funding approval. The non-parametric Mann Whitney U- test was used to compare the number of media reports for breast cancer drugs to non-breast cancer drugs. All of the statistical analyses were performed using Stata, release 9.0 (Stata Corp., College Station, Texas, USA).

## Results

### Time to funding

The first interim analyses of three adjuvant trastuzumab studies were presented at a late- breaking session at the ASCO meeting on May 16, 2005 [12,13]. On July 22 (67 days later), trastuzumab for early stage breast cancer received approval for funding in the province of Ontario. Funding was made available 10 days later on August 1 2005. The peer reviewed publications were not published until October 20 2005, a further 81 days later [8,9].

Table 1 presents the time to funding decision as of August 1 2005 for 18 intravenous and oral anti-cancer agents and indications in Ontario. While funding for trastuzumab was made available within 3 months of release of study results, the median time to funding for 17 other drugs was 31 months (range 14–46). At the time of data censoring (August 1^st ^2005), a funding decision had still not been made for 9 of these 17 indications.

**Table 1:** Time to funding and episodes of media coverage for 18 anti-cancer agents and indications in Ontario as of August 1 2005.

**Drug^ξ^**	**Indication**	**Date Results Announced**	**Date Funded^¥^**	**Elapsed Number of Months^€^**	**Episodes of Media Coverage**	**Number Media Reports Per Month**
Liposomal doxorubicin [14]	Relapsed ovarian	21-05-00	01-10-01	16	0	0
Imatinib (PO) [15]	Unresectable GIST	18-05-01	20-02-03	22	2	0.09
Zoledronic acid [16]	Metastatic prostate	02-06-01	01-01-04	31	1	0.03
Zoledronic acid [17]	Metastatic breast	21-10-01	Ongoing	46	1	0.02
Docetaxel [18]	Neoadjuvant breast	10-12-01	01-08-04	32	3	0.09
Anastrazole (PO) [19]	Adjuvant breast	10-12-01	22-02-05	39	33	0.85
Docetaxel [20]	1^st ^line ovarian	18-05-02	Ongoing	38	1	0.03
Liposomal doxorubicin [21]	Metastatic breast	18-05-02	Ongoing	38	1	0.03
TAC [22]	Adjuvant breast	19-05-02	Ongoing	38	6	0.16
Premetrexed [23]	Mesothelioma	20-05-02	Ongoing	38	7	0.18
Vinorelbine/trastuzumab [24]	Metastatic breast	12-12-02	01-08-05	32	0	0
Letrozole (PO) [25]	Adjuvant breast	05-12-03	Ongoing	20	16	0.8
Capecitabine (PO) [26]	Adjuvant colon	06-06-04	Ongoing	16	0	0
Vinorelbine [27]	Adjuvant NSCLC	06-06-04	01-08-05	14	17	1.21
Paclitaxel [28]	Adjuvant NSCLC	06-06-04	Ongoing	14	3	0.21
Bortezomib [29]	Relapsed myeloma	06-06-04	Ongoing	14	13	0.93
Docetaxel [30]	Metastatic prostate	07-06-04	01-08-05	14	17	1.21
Trastuzumab [12–13]	Adjuvant breast	16-05-05	01-08-05	3	51	17

^ξ^Drugs are intravenous unless otherwise noted and are listed in chronologic order from date of results announced.

^¥^Data was censored at August 1^st ^2005. If no funding decision had been made by this date, time to funding was calculated using August 1^st ^2005 as the cut-off date.

^€^Rounded to the nearest month.

### Media coverage

**Figure 2:**
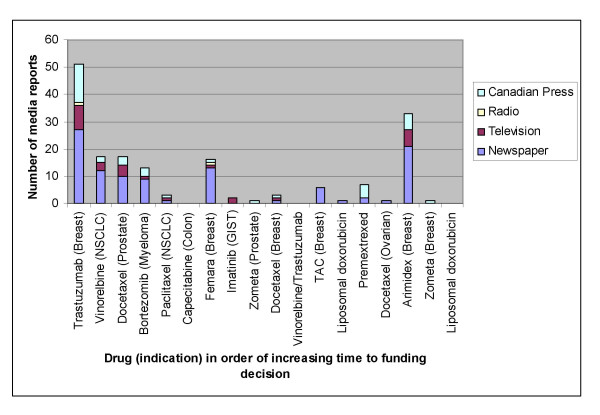
Type and frequency of media reports between release of results and drug funding decision as of August 1, 2005. Drugs are arranged along X-axis in increasing time to treatment funding decision.

**Figure 3:**
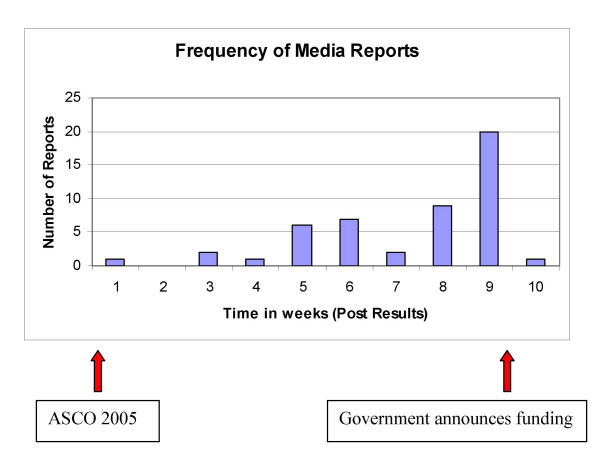
Frequency of media reports for adjuvant trastuzumab.

During the 76 days between the announcement of trastuzumab results and funding becoming available, we identified a total of 51 episodes of media coverage; 27 newspaper, 9 television, 1 radio and 14 Canadian Press items (Figure 2). The frequency of reports followed an upward trend as the weeks passed reaching a maximum on week 9. This coincided with the provincial government announcing approval for trastuzumab for early stage breast cancer (Figure 3).

As shown in Figure 2 there was considerably less media attention for other drugs and indications. After trastuzumab (n = 51) the drug with the most media coverage was anastrozole for adjuvant therapy of breast cancer (n = 33). However with the latter, the 33 media reports were over 39 months translating approximately 1.2 reports compared to 17 reports per month for trastuzumab (Table 1). Other recent major advances in oncology such as adjuvant vinorelbine/cisplatin for resected non-small cell lung cancer (NSCLC) [27] and docetaxel for advanced prostate cancer [30] received considerably less media attention (17 media reports for each) than trastuzumab. Overall, there was a negative correlation where increased media coverage was associated with a reduced time to drug funding approval. (Spearman's rho r = 0.30, p = 0.23, 95%CI: -.84 to 0.23). The median number of media reports for breast cancer drugs (n = 8) was 4.5 compared to 2.5 for non-breast cancer drugs (n = 10) (p = 0.56).

## Discussion and conclusion

In Ontario, the review process for trastuzumab was fast-tracked, meaning that the drug was recommended several months sooner than would normally have been the case. "Our goal is always to get all Ontarians better access to the effective medications and treatments they need," said the Minister for Health and Long-Term Care George Smitherman. "With Herceptin, we were able to speed up, but not compromise, the extremely important review process" [31]. This press release occurred at the tail-end of 9 weeks of mounting media and public pressure. While this was excellent news for breast cancer patients, it raises concern about the speed of funding approval for other agents and indications.

In this report we focus on the potential for media to influence drug funding decisions. Other factors (politics, patient advocacy groups, and industry) have also been implicated in other (non-cancer) drug approval processes. In their review of drug approval time at the United States Food and Drug Administration (FDA), Carpenter and Fendrick found that the time to drug approval was related to staffing levels at the FDA and disease politics (including media coverage and wealth of patient advocacy groups) [32]. The decision of the FDA to postpone the switch of emergency contraception (Plan B) to over-the-counter status was widely attributed to political interference [33]. Finally, it has been suggested that political factors contributed to the "chaos and conflict" surrounding the introduction of adjuvant trastuzumab in UK [34,35]. As depicted in Figure 1 it is unlikely that these external factors operate in isolation; there are likely interdependent relationships between media, politics, industry and patient advocacy, which together may influence drug funding decision-making.

One may expect that speed of drug funding decisions be influenced by the magnitude of clinical effect, the number of patients who could benefit from the treatment, and whether the treatment was curative or palliative. Accordingly, it would not be surprising if a drug for a rare disease with only a small magnitude of benefit in the palliative setting was not funded rapidly, if at all. Clearly this was not the case with trastuzumab which has an impressive 18% absolute increase in 4 year DFS, [12] a 2.7–4% absolute increase in overall survival (at 2 and 4 years), [11,12] and can benefit many women in Ontario with this relatively common condition. Although many of the drugs listed in Table 1 were associated with smaller improvements in patient outcome, the data in support of adjuvant chemotherapy for NSCLC were even more impressive than those for trastuzumab. The JBR.10 study of adjuvant vinorelbine/cisplatin for early-stage NSCLC demonstrated an absolute improvement in 5 year survival of 15%. Despite the impressive result of this Canadian-led study, funding for adjuvant vinorelbine/cisplatin was not available until 14 months after release of study results [27,36].

The relative cost of these agents also does not explain the speed at which funding decisions were reached. Comparing drug acquisition costs alone (figures in Canadian dollars) a course of vinorelbine/cisplatin for resected NSCLC ($2 448) compares very favorably to a course of adjuvant trastuzumab ($45 474) [37]. Furthermore, each of these clinical conditions are common. In the coming year it is estimated that approximately 370 patients in Ontario will receive adjuvant vinorelbine/cisplatin for NSCLC and approximately 1150 patients will receive a course of adjuvant trastuzumab for breast cancer [38].

In the current study we have shown that there was considerably more media coverage for trastuzumab than other anti-cancer therapies. A recent report from the UK suggests that intense media pressure influenced the decision to fund adjuvant trasztuzumab [35]. The reasons for differential media interest are not clear but the contrast is most striking when comparing adjuvant vinorelbine/cisplatin for NSCLC to the trastuzumab experience. This phenomenon has been described in other reports in the literature. In their review of health-related articles in Canadian women's magazines, Hoffman-Goetz and MacDonald found disproportionately fewer articles pertaining to lung cancer than breast cancer relative to the mortality associated with each of these conditions [39]. In a separate report, Otterson has proposed several hypotheses to explain the lower public and media profile of lung cancer. These include: the high mortality of lung cancer which means there are fewer survivors and advocates to lobby on its behalf; lack of prominent spokespeople highlighting the illness; the perception that lung cancer is self-induced by cigarette smoking; and finally the possibility that lung cancer (and smoking) is associated with lower socio-economic status [40]. In contrast, breast cancer has a very large and well organized survivorship population with prominent spokespeople and may be seen as a disease affecting otherwise healthy women.

Regardless of the reason for varying levels of media coverage for different anti-cancer drugs, the degree of media attention may have a significant impact. In the recent Cochrane overview it was reported that health-related media coverage can induce important changes in the delivery of health care [4]. The media clearly plays an important role in the dissemination of health-related information, however it is critical that health care providers and policy-makers recognize the possibility that these reports may present a biased or incomplete picture of the data and may exert undue influence on drug funding decisions.

The current drug funding process in Ontario (CED-CCO) appears (at least in certain instances) to have the ability to generate funding decisions more quickly than its predecessor. However, there are some limitations to this observation. The trastuzumab decision was released as part of a "package" of funding decisions that represented the first assessments made by the group (including vinorelbine for the adjuvant treatment of lung cancer). It is possible that some of the delay in the decision for vinorelbine for lung cancer was due to the initiation of the new priority-setting process. However, the lack of transparency in the current CED-CCO decision-making process makes it difficult to fully understand and appreciate how funding decisions were made, why decisions took the time they did, and the potential influence of external factors such as media attention. Furthermore, the closed decision-making process is contrary to one of the basic tenets of the "legitimate and fair process" described by Daniels [1]. In fact, within Ontario there is currently a move towards a more transparent process with the "Transparent Drug System for Patients Act" that has recently been approved in the provincial legislature [41].

Although the difference in media attention and time to funding between trastuzumab for breast cancer and other anti-cancer agents is striking, this does not establish a causal relationship. The rationing of medical resources is a complex and multifactorial process that relies largely on evidence of clinical benefit. However, this process is subject to external factors including the influence of media, patient advocates, politicians, and industry. The data in this case study serve to illustrate the significant involvement one (or all) of these external factors may play in the debate over priority-setting. An inherent limitation of this descriptive case study is that we are unable to quantify or directly account for the influence of the media or any other external factors. Nevertheless, while the speed of decision for trastuzumab funding in Ontario is laudable, to ensure that priority-setting for cancer therapies is a legitimate and fair process, we must recognize the potential impact of external factors on decision-making and ensure that all new and effective anti-cancer agents are evaluated promptly and in a transparent manner.

## Authors' contributions

All authors contributed to the acquisition and analysis of data presented in this study and the writing of the manuscript. All authors have given final approval to the manuscript. There was no source of funding for this study and all authors declare no conflicts of interest.

## Pre-publication history

The pre-publication history for this paper can be accessed here:


